# Critically ill patients with 2009 H1N1 infection in an Indian intensive care unit

**DOI:** 10.4103/0972-5229.78236

**Published:** 2011

**Authors:** Vivek B. Kute, Pankaj R. Shah, Manoj R. Gumber, Aruna V. Vanikar, Hargovind L. Trivedi

**Affiliations:** Department of Nephrology and Transplantation Medicine, Laboratory Medicine and Transfusion Services and Immunohematology, IKDRC-ITS, Ahmedabad, India; 1 Department of Pathology, Laboratory Medicine and Transfusion Services and Immunohematology, IKDRC-ITS, Ahmedabad, India

Dear Editor,

We read with great interest the report by Chacko *et al*.[1] The authors reported an interesting experience on 2009 H1N1 infection. World Health Organization’s (WHO) “new” Influenza A (H1N1) Case Summary Form for case-based data collection is to be used to obtain important information to determine severity and clinical characteristics of the cases infected with “new” Influenza A (H1N1).[2] According to Centers for Disease Control and Prevention (CDC), the use of a standardized data collection instrument will aid in uniform data collection of geographically dispersed cases and for clinical comparison to assess changes in viral pathogenesis and clinical course over time.[3]

We believe that with some additions, according to WHO and CDC case summary form, as mentioned below, scientific value and contribution of the article may increase in prospective study.[1–3]

The details to be included are as follows. Vaccination history with seasonal influenza, H1N1 or pneumococcal vaccine? History of use of Oseltamivir as prophylaxis or treatment before admission? Oseltamivir timing of initiation, dosing, duration, adverse effects and dose modification in acute kidney injury (AKI). History of epidemiologic risk factors could have been included. Antiviral treatment is most likely to provide benefit when initiated within the first 48 hours of illness. CDC and WHO note that some experts have advocated increased (doubled) doses of Oseltamivir and that hospitalized patients with severe infections might require longer treatment courses (e.g., 10 days). Since Oseltamivir is primarily excreted by kidneys, dosing must be modified for renal insufficiency.[4]

The pandemic of H1N1 influenza A infection appears to involve sustained human to human transmission. Therefore, what infection control measures were implemented for 2009 H1N1 influenza in healthcare settings, including protection of healthcare personnel, family member and close contact, and in hemodialysis settings including Oseltamivir prophylaxis. What was the secondary attack rate or bacterial super-infection rate?

Possible etiology of AKI is multifactorial, resulting from acute tubular necrosis due to shock, hypoxemia of acute lung injury, hypoperfusion, renal vasoconstriction, and rhabdomyolysis.[5]

Multicenter, prospective, randomized, controlled clinical trials should be carried out to identify the effectiveness of early Oseltamivir treatment, protection offered by having undergone seasonal influenza and H1N1 vaccination and to identify potential poor prognostic indicators in the future.

Pooling of data, according to uniform and a standardized WHO, CDC case summary form, from various centers across the world, would certainly help a better understanding of similar outbreaks and help administrators plan for such disasters in the future.
